# Chemical Researches on the Human Blood

**Published:** 1838-10

**Authors:** 


### Art. X.

1. *Etudes Chimiques sur le Sang Humain.* Par Louis-René Le Canu, de Paris, d.m.—*Paris*, 1837.

*Chemical Researches on the Human Blood.* By L. R. Le Canu, d.m.—*Paris*, 1837. 4to. pp. 128.

2. *Nouvelles Expériences sur le Sang.* Par M. P. Denis, de Commercy. (*Archives Génerales de Médecine, Fevrier*, 1838.)

*New Experiments on the Blood.* By M. P. Denis, of Commercy. (*Arch. Gén. de Médecine, for February*, 1838.)

There are few subjects which have more deservedly engaged the attention of modern chemists than the analysis of the blood in health and disease. If the results hitherto obtained, are of too vague a nature to allow them to be applied to the improvement of the practice of medicine in the way anticipated by the experimenters, this is rather to be ascribed to the extreme difficulties which beset the investigation than to any want of zeal or skilfulness on their parts. Ample materials are before us; but these materials require a master-hand to mould them into a proper form. The differences in the results of different chemists must be reconciled; in making extensive and important generalizations, we must not be content to proceed with mere average ratios; and, above all things, the characters of blood in its most normal or healthy condition must be indisputably settled. M. Le Canu, the author of the thesis placed at the head of this article, whose name is already well known to the chemists and physiologists of this country, appears to be fully aware of the difficulties which remain to be overcome. To make our knowledge practically available, we must determine, in the healthy adult, the number, nature, and proportion of the constituent principles of the blood which enters into, as well as of that which passes out of the lungs. The liquid transmitted through the jugular, femoral, and subclavian veins, in other words, that which comes from the head and extremities, should be attentively compared with the contents of the vena portæ, the hepatic veins, and of the left subclavian vein, after it has received the chyle from the thoracic duct. The arterial must be compared with the venous blood in individuals of the two sexes, of different ages and temperaments. A similar series of experiments should likewise be instituted on the blood of persons labouring under different forms of disease. It is easy to understand that all the conditions here mentioned may give rise to important modifications in the composition of this fluid.

M. Le Canu has endeavoured in his thesis to give us a true picture of the present state of our chemical knowledge on this subject. Before we express an opinion of the manner in which he has executed this laborious task, we consider it necessary to lay before our readers a general outline of his researches; premising that, in doing so, we are furnishing them not only with the most recent, but with the most trustworthy account of the properties of the blood.

The thesis is divided into four parts: in the first part, we have an examination of the nature and mode of distribution of the immediate principles of healthy venous blood; in the second, the determination of the proportion of these principles, under the different conditions of age, sex, temperament, and kind of food; in the third, the arterial is compared with the venous blood; the properties of this liquid as it is found in the capillary system, vena portæ, and placenta, are then examined. Finally, in the fourth, we have a summary of the pathological changes which it undergoes in certain states of disease, more especially in icterus, cholera, chlorosis, and affections of the heart.

Chemists have generally selected blood for analysis from the veins at the bend of the arm; since, as the arterial blood of the hand traverses no secreting organ before arriving at this part, it may be taken as the type of venous blood in its greatest possible state of simplicity. No less than *forty-five* different constituent parts are enumerated as entering into the composition of this liquid; and, to facilitate the labours of those who are disposed to follow out its minute history, copious references are given opposite to each constituent. But, as the author wisely observes, the existence of this large number of bodies in venous blood is far from being satisfactorily established. The only chemist of repute who has met with manganese is M. Denis, to whose experiments we shall presently have occasion to refer; but he himself admits, what all will easily believe, that this metal may have been accidentally introduced during the analysis. M. Le Canu places more confidence on the discovery of copper by M. Sarzeau, (*Journal de Pharmacie,* t. xvi., p. 505;) but, at the same time, he does not seem disposed to admit it as a regular constituent. Notwithstanding the acknowledged ability of M. Sarzeau as an analyst, since no other chemist has found copper in the blood, we are justified in thinking that he may have been deceived: for the discovery of copper even in the small proportion of $\frac{1}{1000000}$th part of an *organic* liquid containing it, is not attended with any great difficulty. This experimentalist stated that he found copper in a great number of organic substances, as in the varieties of corn and grain, in flour, bread, cheese, meat, and most kinds of food, a statement, which has been more industriously circulated than closely examined. Our own experiments, both with regard to the blood and some of the other substances mentioned, lead us to declare, either that some unperceived error in spite of the greatest precautions must have crept into them, or that the blood and the other bodies mentioned, do not contain copper. The hydrosulphuret of ammonia, the acetate and benzoate of soda, discovered by Prevost, are properly considered to have resulted from the decomposition of the liquid under analysis, and have no claim, therefore, to be regarded as independent constituents. The hematic acid, which, according to Treviranus, is combined with iron, has been proved by Engelhart to be the hydro-sulphocyanic acid: and that this was plainly a result of decomposition, is proved by the fact that it was only discoverable after the carbon of blood had been heated to redness with soda. The osmazome of Denis, the *extractive* matters of Berzelius, and the muco-extractive matter of Marcet, are undefined and complex products: besides, these different names appear to be applied to substances essentially the same. The older chemists thought that gelatin was a constituent of the blood; but the researches of Berzelius, Brande, Bostock, and Marcet, have proved that this is an error.

"The combination of albumen and soda, usually known under the name of serosity, was formerly mistaken for gelatin: but there is this difference between them. A solution of gelatin, exposed to a voltaic current, undergoes no change; while the gelatinous-looking serosity is instantly separated into albumen and soda. Besides it is now generally acknowledged that gelatin is not a distinct animal principle; but a mere product of the reaction of boiling water on skin, cartilage, and other substances." (*Le Canu,* p. 14.)

Some dispute has existed as to the presence of urea in the blood. The real state of the question seems to be this. MM. Dumas and Prevost, in the first instance, demonstrated that urea existed in the blood of those animals whose kidneys had been extirpated; but neither they, nor Tiedemann and Gmelin subsequently, could detect the smallest trace of urea in the blood of animals placed under ordinary circumstances; although, according to the latter chemists, urea may be detected in the proportion of $\frac{1}{250}$th even in operating on a very small quantity of blood. M. Le Canu observes:

"I have not succeeded in discovering urea in one thousand grains of human serum; but I have observed that an aqueous solution of an alcoholic extract of this serum, first dried in a vapour-bath, and then digested in ether, gave out during the evaporation a strong urinous odour; still the solution evaporated to the consistency of a syrup, cooled and mixed with an equal bulk of strong nitric acid, did not furnish a trace of the nitrate of urea." (p. 15.)

This we think is conclusive of the absence of urea from *healthy* blood: for it is impossible to rely upon an odour resembling that of urine as evidence of the presence of urea. This principle is well known to be inodorous.

Passing over a few other unimportant substances, the existence of which as constituents is doubtful, M. Le Canu arrives at the conclusion that there are at least twenty-six bodies present in the blood. These are:

"Oxygen, nitrogen, carbonic acid, iron, the muriates of soda, potash and ammonia, the sulphate of potash, the *sub*-carbonates of soda, lime and magnesia, the phosphates of soda, lime, and magnesia, the lactate of soda, fixed fatty acids combined with soda, a salt formed by a volatile fatty acid, fatty phosphorescent matter resembling the cerebral substance (cerebrine), cholesterine, seraline, fibrin, albumen, yellow, and red colouring matters, extractive matters, water." (p. 16.)

Under the very indefinite term of "*extractive* matters," are comprised: the osmazome and cruorine of M. Denis, the extractive matter of Berzelius and Marcet, the serosity or compound of albumen and soda of Brande and Le Canu. It is very properly remarked, that until the true nature of these substances has been determined, no mischief, but much good will arise, from considering and classifying these nominally different substances under one head.

Probably the long list of substances above given might be advantageously reduced. Thus, with regard to the salts, we doubt whether any with an ammoniacal base can be truly said to be a constituent of healthy blood. Is it not much more probable that this alkali should have resulted either from spontaneous decomposition before analysis, or have been actually produced in the processes required to determine its presence? Again, how can it be said that the sub-carbonates (carbonates) of lime and magnesia, exist free in the blood? If we admit that the serum owes its alkalinity to the presence of carbonate of soda, we cannot extend this admission to the insoluble carbonates of lime and magnesia; and we think it a point far from being satisfactorily settled, as to how far the different salts of the blood have really an independent existence in it. Where, as in general, the process by incineration is followed for their detection, the fact that sulphur, phosphorus, and carbon exist in the organic principles of this liquid, must tend to render it probable that the acids of these salts may be, if not entirely, at least in part, accidentally produced.

Our author does not propose to examine the chemical properties of the different bodies which he enumerates as constituents: for an account of these, he refers to the different and well known writings of other chemists. Nevertheless, the state of the iron in blood, and the singular but little understood connexion existing between it and the red colouring matter are points which are sufficiently important and interesting to call for a few remarks.

The presence of iron in blood is now placed beyond all dispute; but its proportion has been considerably exaggerated. Thus, Menghini, who discovered it, supposed that at some future day, nails, swords, and all kinds of instruments might be manufactured from it. "Non desperaverim ex humano sanguine et clavos et enses, et instrumenta omni genere cudi posse." One of our own countrymen, Dr. Good, asserted, that there was enough iron in the blood of forty men to make a good ploughshare: (*Book of Nature*, vol. i. p. 361;) and Deyeux and Parmentier broached the idea that medals might be struck to the memory of celebrated men from the iron obtained from their blood! We shall see, however, that the quantity is in by far too small a proportion to realize these ingenious speculations. One fact seems now to be well established, that that portion of blood which is commonly called the clot alone yields this metal. Brande asserted that he obtained an equal portion of iron from serum: but Berzelius and Engelhart subsequently proved that there must have been some error in his experiments. Berzelius, however, admits that there are faint traces of iron in the ash obtained by the incineration of fibrin and albumen. But is the iron inherent in and intimately combined with the colouring matter, or is it merely loosely mixed with it in the state of oxide, phosphate, or in the form of an organic compound? On this point, some of the best authorities are at issue. Berzelius, Engelhart, and Le Canu, separate the colouring matter from blood by different processes; but each has found in his experiments that the iron came exclusively from the colouring matter, and they consider that it is in an intimate state of combination with it. On the other hand, Brande, Vauquelin, and Sanson, by processes peculiar to themselves, assert that they succeeded in separating from blood colouring matter free from iron; they therefore contend that the iron is not combined with it. It will not be difficult to reconcile these discrepancies. Berzelius has shown that the colouring principle, as obtained by Brande, does yield peroxide of iron on incineration; and indeed this chemist himself admits, that he always found *traces* of that metal in the incinerated residue, although, at the same time, he denies that it is essentially united to the colouring matter. The inferences of Vauquelin and Sanson are set aside by the fact, that they did not operate on the calcined residue. In taking this precaution, Berzelius and Le Canu both detected iron in a proportion equal to that obtained from their own experiments; and by pursuing the process of Sanson for extracting the colouring matter, our author invariably succeeded in demonstrating in it the presence of the metal. We can then come to no other conclusion, than that the iron and colouring matter of blood are intimately combined; and cannot, by any processes short of the utter destruction of the organic matter, be separated from each other. The author then details six experiments in which he extracted the colouring principle from blood by six widely different processes; and yet in each case there was no difficulty in determining the presence of iron in it. We have only room for two of these, which we quote, as they appear to us to show in the most satisfactory manner the intimacy of the union of these two bodies.

"*Experiment* 1. Warm alcohol was digested on dried blood. The alcohol became strongly coloured; and, on cooling, an abundant red deposit took place. This deposit washed, dried, and calcined, yielded an ash very rich in peroxide of iron.

"*Experiment* 3. The blood of the ox was boiled with alcohol containing a few drops of sulphuric acid. I obtained, 1st, an abundant colourless residue, the ashes of which were entirely free from iron; 2d, brown-coloured solutions, which an excess of ammonia changed to a red colour without precipitating a particle of oxide. In these solutions none of the reagents for iron indicated the slightest trace of that metal; while by evaporating them and incinerating the residue, it was clear that they contained it in large quantity." (p. 23.)

But there is great reason to doubt, as the author justly remarks, whether by any of the processes usually resorted to the colouring matter of blood is obtained in a pure state. Thus, according to the method of Berzelius, namely, that of drying slices of the clot on bibulous paper, and the processes proposed by Brande and Vauquelin, the colouring matter must be always united to more or less fibrin and albumen. The plan adopted by Engelhart was to dilute the concentrated solution of colouring matter with so much water, that on heating it to a certain temperature this would alone separate in coagula; while the albumen from the large quantity of water present would, it was supposed, remain uncoagulated by heat. But Berzelius proved that a portion of albumen always separated in a coagulated form with colouring matter; and if any albumen were to be detected after the process in the residuary liquid, this was to be ascribed to the dissolving power of the alkali set free during the experiment. This fact is certain: we can never by the mere washing of a clot in water obtain colouring matter without albumen; therefore experiments, performed, as they usually are, upon such a compound, are not to be relied on as setting forth the properties of the pure colouring principle of the blood. The presence of albumen in the colouring matter, obtained by the different processes above mentioned, was easily demonstrated by Le Canu, in simply washing them with hot alcohol acidulated with sulphuric acid. (p. 26.) Even the principle which he had previously described as *globulin* was thus proved to be a mixture of colouring matter and albumen, but the latter was in less proportion than usual. The colouring principle which was procured by Sanson, although free from albumen, he regards as an altered product, from the means used to procure it. From these facts, it is easy to understand why the properties of this important constituent of blood have been so differently described by different observers. Indeed, in comparing the accounts of some chemists, it would hardly be supposed that they were describing a substance which, like other independent animal principles, should possess certain distinctive and uniform characters.

M. Le Canu proposes to substitute the name of *hæmatosine*, which was first given by Chevreul to the colouring matter of blood, for *globulin*, subsequently suggested by himself. That is the term which is now, indeed, generally applied by chemists to designate this principle; but we must here premise that the hæmatosine of Le Canu differs widely in its properties from the hæmatosine of other experimentalists. We must refer to the work itself for a full description of the process for obtaining pure colouring matter. The principle of separation is simply this: The blood, deprived of its fibrin, is treated with sulphuric acid until the whole sets into a brown mass. To this alcohol is added, and the mass compressed in linen, by which the water of the blood is removed. The brown residue is now digested by divided portions in boiling alcohol, slightly acidulated, if necessary, until the last portions of alcohol are no longer coloured. By this we obtain a dense white residue,—a compound of sulphuric acid and albumen,—and several alcoholic solutions of a reddish brown colour, holding, among other matters, the red colouring principle. The alcoholic solutions are supersaturated with ammonia, filtered and evaporated to dryness. The residue is a mixture of colouring matter with saline and fatty substances; these latter are separated by repeated digestions in water, alcohol, and ether. The chief properties of hæmatosine thus obtained are the following:

"It is solid, inodorous, insipid, of a dull brown or a reddish black colour, with almost a metallic lustre. It is insoluble at any temperature in water, alcohol, ether, and oil of turpentine, unless these menstrua be combined with a few drops of ammonia, potash, or soda, when it forms a solution of a blood-red colour. By long contact with alkalies, or by boiling in water, it becomes changed in colour and properties; for it is now no longer dissolved by ammoniacal alcohol. Chlorine passed through water holding it mechanically suspended entirely destroys it. White flocculi subside insoluble in water, but soluble in alcohol, while the supernatant liquid will be found by the application of reagents to have become impregnated with iron. The sulphuric, nitric, and muriatic acids act powerfully on it, and entirely alter its properties. When a portion is deflagrated with nitrate of potash, neither sulphuric nor phosphoric acid can be detected in a solution of the residuary salt; facts which prove that hæmatosine contains neither sulphur nor phosphorus. When incinerated, it yields a reddish-coloured ash destitute of alkaline reaction, forming a yellow solution without residue in muriatic acid, on which the ferrocyanate of potash, the hydrosulphuret of ammonia, and the infusion of galls, act as upon a solution of permuriate of iron. The muriatic solution evaporated to dryness yields a solid hyacinth red substance, not a white residue as it would do if it contained phosphate of iron. These experiments show that the ashes of colouring matter consist exclusively of peroxide of iron." (p. 30, *et seq.*)

The author found that one hundred parts of hæmatosine obtained from the blood of two females, the one aged twenty-eight and the other eighty-three years, as well as of two male subjects aged twenty-nine, yielded in each case ten parts of peroxide of iron, which are equivalent ($\frac{28}{40}$) to seven parts of metallic iron. The hæmatosine procured from the blood of mammalia, birds, reptiles, and fish, was found to possess the same physical and chemical properties as that derived from the blood of man. M. Le Canu thence takes occasion to animadvert, and in our opinion very justly, on the absurdity of attempting to distinguish the blood of various species of animals effused on linen and clothes from that of a human being. (p. 38.) Although there was no difference in the general properties of hæmatosine, whatever the animal from which it was taken, yet the proportion of iron yielded on incineration was found to vary. Thus while, as we have seen above, one hundred parts of human hæmatosine yielded ten parts of peroxide of iron, the same quantity of hæmatosine of the ox yielded in one experiment 12.85 parts, and in a second 12.67: while this quantity of hæmatosine from chickens' blood gave only 8.34 parts of peroxide. Thus the quantity of iron, although it seems pretty uniform in regard to individuals of the same species, is subject to variation in individuals of different species, and, *à fortiori*, of different classes.

But in what state is the iron combined with the colouring matter? This is a question which has for some time past exercised the ingenuity of chemists, and the answer to which still remains in a great degree a matter of speculation Deyeux and Parmentier considered that the metal was in the state of peroxide, suspended or rendered soluble by free alkali. Fourcroy and Vauquelin afterwards imagined that the peroxide was combined with phosphoric acid as a basic phosphate: but Berzelius showed that this was not the case: since the subphosphate of iron is not soluble in serum or any modification of albumen, whether an alkali be added or not; and that the colour of the blood could not depend upon the presence of subphosphate or peroxide of iron, was proved by the fact that artificial mixtures containing either of these bodies possessed only a rusty colour, and by no means the bright red tint of the blood. Le Canu is inclined to adopt the opinion of Berzelius, that the iron is in the state of *pure metal* in the blood forming one of its elements, just as phosphorus united to oxygen, hydrogen, and carbon, forms one of the elements of the fatty matter of the brain. (p. 36.) The grounds for this opinion are: if the iron were in the state of oxide, it ought to be separable from the hæmatosine by the muriatic and other mineral acids, which it is not. It ought also to be susceptible of detection by the common and delicate reagents for iron, whereas we know that in a strong solution of hæmatosine these are without effect. With perhaps only one exception the iron cannot be detected in blood prior to incineration. This exception refers to the experiment of Engelhart of passing chlorine gas through a mixture of hæmatosine, in which case the colouring matter is destroyed while muriate of iron is formed and held in solution. It has been contended that this is an additional proof of the iron being in the state of metal since chlorine has no tendency to unite to the oxide of a metal; but Fromherz answers this statement by supposing that the chlorine, in destroying the organic matter, passes to the state of muriatic acid, sets the oxide of iron free, and then combines with it. Rose made artificial mixtures of oxide of iron with different kinds of organic matter; and proved that in these combinations, the tests for iron wholly failed to detect the presence of the metal. Therefore, he argued the iron in blood might be in the state of oxide combined with organic matter, and not be susceptible of detection by tests, owing to that very combination. There is no doubt of the correctness of Rose's experiments: but Berzelius overthrew the conclusion derived from them, by establishing that acids had the power of readily dissolving out the oxide of iron from all such artificial compounds; whence it followed that the analogy between the state of the iron in them and in hæmatosine, wholly failed. Although it is not capable of demonstration, there seems therefore to be every reason to believe that the iron in blood is united in the form of *pure metal* to the ultimate elements of hæmatosine, just as it is united to carbon, nitrogen, and hydrogen in ferrocyanic acid. In both cases it is withdrawn from the operation of ordinary reagents, while in each it is easily susceptible of detection after incineration.

But in whatever state we may presume the iron to exist, there is no reason to suppose that the colour of the blood is due to that metal either in the form of oxide or salt. It is an essential constituent of hæmatosine, and forms, as we have seen, about seven per cent. of that principle; but the colour of the blood is wholly different from that which any salt of iron is capable of giving, even supposing that it were in sufficient quantity. There can be no doubt that the colour of the blood is due to an organic red colouring principle, similar to that of madder or cochineal, which forms the great bulk of the hæmatosine.

Some chemists have thought that there existed a strong analogy between hæmatosine and albumen, but our author shows that there are differences, of which the following are the most important: "Hæmatosine is soluble in ammoniacal alcohol, albumen is perfectly insoluble in this menstruum. Hæmatosine is insoluble in the acetic, muriatic, and sulphuric acids, while albumen is dissolved by them. Lastly, hæmatosine leaves one-tenth of its weight of an ash consisting of the peroxide of iron, while albumen leaves on incineration no (?) traces of iron." (p. 37.)

After some general remarks on the manner in which the chief constituents of the blood are disposed, as well as on the variety of opinions existing respecting the form, size, and disposition of the globules, the author proceeds to detail his own observations on the fatty principles contained in the serum, chiefly the margaric and oleic *acids*, and on the constitution of the globules.

M. Le Canu adopts the old opinion of Home, Prévost, and Dumas, that fibrin has no independent existence as such in the blood; but that it is an essential constituent of the globules, which he believes to be formed externally of a transparent envelope of fibrin, and internally of a mixture of albumen and hæmatosine. (p. 53.) Thus then, according to this view, the blood is divided into two parts, serum and globules; but the whole of the crassamentum is not formed of globules. This mass locks up within its spongy texture a quantity of serum which must be drained away from it by means of bibulous paper. We then have a residue representing the weight of the globules. The mean proportions in 1000 parts of healthy blood are thus given:

| | Le Canu. | Prévost and Dumas. | M. Denis. |
|---|---|---|---|
| Serum . . | 869.1 | 870.8 | 876.9 |
| Globules . . | 130.9 | 129.2 | 123.1 |
| | 1000. | 1000. | 1000. |

These, it is to be observed, are only average results. In no two experiments will the proportions be probably exactly the same; since the degree to which the crassamentum is drained, and the difficulty of separating from it the whole of the loose serum must make a difference. This will likewise account for the above numbers not corresponding: but the difference there is not very material, although in *individual* instances M. Denis found it very great. Thus it is stated in his paper (p. 179,) that the maximum proportion of globules in 1000 parts of blood reached 173.1; while the minimum proportion was so low as 64.4. We must refer to a table by M. Le Canu (p. 62,) to show that there are great variations in the proportion of the globules, depending in some degree upon the *age* of the subject from whom the blood is taken.

The proportion of hæmatosine in the globules is determined by the process already given. M. Le Canu has not found it to form more than 2 per cent. of the globules; and therefore the colouring matter of blood forms very little more than 2 parts in 1000 of that liquid. This will appear extraordinarily small, especially when we compare it with the statement of M. Denis, (*Archives*, p. 173,) that the hæmatosine forms 18 parts in 1000 of blood; but the discrepancy is removed when we bear in mind that the colouring matter of M. Denis contains albumen, while that of Le Canu is free from this principle.

M. Le Canu analyzed the globules by receiving fresh blood in a saturated solution of sulphate of soda. This prevented coagulation, and kept the globules in his view with their envelopes entire. "After a few hours, the liquid will be found separated into two parts, the upper having a faint reddish tint, while the lower is thick, of a blood-red colour, and by slight agitation rises in the form of small pearly looking globules." (p. 50.) By filtration the supernatant liquid is easily separated; and is then found to consist of sulphate of soda and serum. It has all the characters of a solution of albumen in that salt. "The deposit when quite drained has the consistency of honey. When digested in alcohol, acidulated with sulphuric acid, it surrenders a very small portion of hæmatosine and leaves an abundant white residue, having all the characters of sulphate of albumen." (p. 51.)

This then proves that the globules contain a large quantity of albumen. The next observation seems to bear out strongly the view of the author, that the fibrin is not a substance freely diffused or dissolved in the blood, but really a constituent of the globules. "The deposit treated with a saturated solution of the sulphate of soda is not dissolved, nor is the saline solution in the least coloured by it. But if the mixture be strongly agitated, a blood-red liquid is obtained; and there is a residue formed of a white membranous looking substance." (ib.)

That this substance is not albumen is proved by its entire insolubility in water: that it is really combined with the colouring matter, either as nucleus or envelope, seems to be well established by the fact that they are not separable from each other except through mechanical agitation or trituration; although the colouring matter itself is proved by this experiment to be easily taken up by the saline solution. For this reason the author considers that fibrin must form the envelope of the globules; since if the colouring matter (hæmatosine and albumen) were external, why should not the solution dissolve it? The fibrinous envelope protects it until this is mechanically destroyed; then it escapes and is dissolved. This, it is true, is an assumption, but it is one which appears to us to be well warranted by the results of the experiment.

The effect of water is singular:

"When water is added to the blood red deposit it is absorbed; and the deposit swells out to a dark red jelly or clot. If the proportion of water be increased, the jelly disappears entirely; and while the liquid assumes and retains a red colour, there slowly separates from it a white membranous matter, which is known to be fibrin by the following characters: It is insoluble in hot and cold water, in alcohol and ether, but little soluble in ammonia and acetic acid, insoluble in water saturated with sulphate of soda, but taken up, on the contrary, by a solution of nitrate of potash." (p. 52.)

We do not consider our author quite correct here: fibrin is remarkable for its solubility in acetic acid; and so far as our observation extends, it is taken up in only very small proportion by a saturated solution of nitrate of potash. He admits that he owes a knowledge of this last property to M. Denis, who, as we shall have occasion to see, is remarkable for the boldness of his assertions.

M. Denis, who holds that there is no difference between albumen and fibrin, contends that fibrin is held dissolved in the blood; and he considers that he has discovered the secret of the solubility of this principle. He asserts that the salts in the serum render it soluble, so long as it circulates through the living vessels. (p. 173.) Thus then in the view of this gentleman, the serum of blood is nothing more than a saline solution of fibrin, and fibrin and albumen are identical. (p. 176.) Insolubility in water, he observes, is the natural character of albumen, and when we find it dissolved this must be ascribed to the presence of saline matter. We shall now be prepared for M. Denis's explanation of the cause of the coagulation of blood when removed from its vessels.

"The serum while within the vessels is naturally supersaturated with albumen, which it is continually depositing in the round of circulation. For the same reason, it must deposit that principle equally when removed from its vessels. It is in this that essentially consists the phenomenon of coagulation." (*Denis*, p. 180.)

M. Denis is the first chemist whom we have met with who has contended that fibrin was identical with *uncoagulated* albumen. Whether we admit that fibrin is held dissolved in the blood of the living body or not, it is impossible to consider that substance as identical with the albumen of serum, or to suppose for a moment that the small quantity of saline matter united to albumen gives to it its property of solubility in water, and constitutes the difference between them. Many chemists have occupied themselves in endeavouring to establish the identity of fibrin and *coagulated* albumen; but with singular inconsistency they have, at the same time, in other parts of animal chemistry, divided what might be fairly, and is generally, regarded as one substance into many others. We ought to remember that a similarity of properties does not constitute identity, and that these attempts to enlarge or diminish the number of bodies where there is so little necessity, are always attended with considerable mischief. We do not mean to assert that there are any striking chemical differences between *coagulated* albumen and fibrin; but in our view, the fact of albumen existing in two states both in and out of the living body, i. e. as solid and liquid, while fibrin is only known to us as a solid out of the body, as also that acetic acid has a very different solvent action on fibrin and coagulated albumen, are differences far greater than exist among many organic substances which are commonly regarded as distinct. M. Denis proceeds to prove his propositions by experiment. He says that "if the fibrin of blood obtained by washing the clot in linen be digested in a solution of nitrate of potash for twenty-four or forty-eight hours or even longer, according to the proportion of salt present, it will be dissolved. The new product will resemble serum or white of egg. It will be precipitated by bichloride of mercury and alcohol, and will coagulate at a temperature of 170°. If this saline solution of fibrin be diluted by a large quantity of water, the fibrin will gradually be precipitated, and reacquire its orginal properties." (*Denis*, p. 174.)

Being struck with the novelty of this experiment we resolved to repeat it, and employed recently prepared fibrin of blood with a pretty strong solution of nitrate of potash. Although the author says nothing about temperature we employed both a *hot* and *cold* solution, digesting and macerating for four days to be quite certain of the result. The liquids were then filtered, and the greater part of the fibrin employed was unacted on and left on the filter. A solution of chlorine which we find to be the most delicate precipitant of fibrin dissolved in acetic acid, gave a faint opalescence, showing that a very minute portion had been taken up; but neither the bichloride of mercury nor a boiling heat had any effect on the filtered liquid. Alcohol certainly gave a precipitate as it always will in a pretty strong solution of nitre, a fact not noticed by M. Denis, although it could not fail were his statements borne out by experiment to complicate the result. In another trial we allowed the fibrin to digest in a cold solution of nitre after very gently heating it for *fifty days;* but *it was not dissolved.* We therefore think ourselves warranted in rejecting both the experiment of M. Denis, and the hypothesis which he has formed from it. But as nitrate of potash is not contained in the blood, he felt himself obliged to advance a step further than this. We are given to understand that he actually incinerated a portion of blood, then obtained from the carbonaceous residue a solution of the saline matter, concentrated the solution by evaporation until the salts amounted to about from $\frac{7}{1000}$ to $\frac{10}{1000}$ths; and by means of this liquid dissolved about $\frac{75}{1000}$ of fibrin, thus forming an artificial serum. (p. 175.) For reasons above given we put no confidence in this statement.

Thus then, if fibrin be dissolved in blood while circulating in the living vessels, this is certainly not proved by the experiments of M. Denis; far less is it established that the albumen of serum is identical with the solid fibrin obtained by washing the clot. M. Denis's explanation of the cause of coagulation cannot then be admitted; but even allowing to him all that he requires it is impossible to adopt his view of this phenomenon. What physiologist will admit that the influence which leads to the deposit of albumen or fibrin by this liquid in the *living* body, is identical with that which leads to the separation of these two principles in blood after it is drawn from the body? According to this view, life takes no share in the phenomena of separation in the living system; but it is a simple mechanical result of the supersaturation of the blood with albumen (fibrin)!

To return to M. Le Canu. Many of our readers may have observed that the liquid obtained by washing a clot of blood in an excess of water, is in the first instance clear and of a deep blood-red colour; but after a time a whitish-red deposit takes place in it. This cannot be albumen, since that is suspended with the colouring matter; but it must be the fibrin separated during the process from the globules, and which Le Canu believes to form the envelopes. This will explain the effect of water on the red deposit obtained by our author. The globules are destroyed by the process of washing, or even by simple digestion in an excess of water, and the fibrinous envelope being insoluble falls to the bottom of the vessel after a longer or shorter time.

M. Le Canu, it will be perceived, is at issue with other chemists chiefly on the point whether fibrin be held dissolved in the blood or not. His opinion of that principle being an essential constituent as a solid envelope of the globules, certainly appears to us to receive support from his observations and experiments. Thus he contends, the globules unless agitated are permanent in saline solutions but are destroyed by water. Hence the globules are suspended in serum without rupture because it contains saline matter. They disappear on the addition of water to blood because the strength of the saline solution is thereby diminished; the envelopes are ruptured and the colouring matter and albumen taken up by the water. Again, it is impossible to obtain separately serum and globules from blood which has once been violently shaken, and this liquid does not coagulate when it has been well beaten or agitated. In both of these cases the facts are explained by the constitution of the globules being destroyed. One point here occurs to us which seems to require explanation. The author employed in his experiments a *saturated* solution of sulphate of soda; but the serum is far from being a saturated solution of saline matter. Besides the most abundant salts in the blood are, upon his own showing, the muriates of potash and soda; and he states he found that a solution of common salt tended to *dissolve the colouring matter* of the globules much more rapidly than the sulphate of soda. Is there not some discrepancy here? Is the author at least not bound to show that an artificial mixture, resembling serum in the *nature* as well as in the *proportion* of its saline contents, has no more influence upon the globules than ordinary serum? The permanency of the globules in this liquid cannot obviously be referred to its saline contents before some such experiment as this has been successfully performed. We put it to the author, whether it be physiologically or chemically accurate to draw an inference respecting this assumed property in serum, from an experiment made upon a *saturated* solution of a particular salt which does not exist in that liquid?

M. Le Canu, although he believes fibrin to be solid in the blood, acknowledges that a coagulable principle occasionally separates from that liquid in a solid form. The nature of this he does not attempt to explain; but he imagines that, under certain conditions of the economy, albumen may acquire the property of becoming spontaneously coagulable. The formation of a buffy coat appears also to offer a difficulty to his view of the constitution of the globules. He considers that this may be due to a similar cause; to the production of a substance analogous to a false membrane on the surface of the crassamentum, or to an increased formation of fibrin. We do not think, however, that this explanation removes the difficulty; for the section of a clot with a buffy coat shows that the colour is gradually shaded off, from the almost colourless surface to the under part, where it is most intense: a fact which seems to prove that the colouring matter is distinct from the fibrin, and is capable of falling through it while consolidating, if the process take place slowly. Besides, those lengthened masses of pure fibrin often found in the heart and great vessels after death cannot have their origin thus explained.

For the method of analysing the blood, we must refer our readers to the work itself; but we think it proper to subjoin the mean result of the analysis of venous blood by the author, as his views are somewhat peculiar respecting its constitution:

*Analysis of Venous Blood.*

| | |
|---|---|
| Water . . . . | 790.3707 |
| Saline and fatty matters . | 10.9800 |
| Albumen . . . | 67.8040 |
| Globules . . . | 130.8453 |
| | 1000. (p. 59.) |

Our readers will here remark that neither fibrin nor colouring matter is mentioned as a constituent of the blood, while the quantity of albumen is less than we find commonly stated. These facts are explained by referring to the author's views on the composition of the so-called *globules*. These are formed of albumen in very large, and of fibrin and hæmatosine in very small, proportion. Thus the following is his analysis:

| | |
|---|---|
| Fibrin . . . . | 2.9480 |
| Hæmatosine . . | 2.2700 |
| Albumen . . . . | 125.6373 |
| | 130.8453. (p. 60.) |

Without this index, we should be at a loss to reconcile the author's analysis with the results obtained by other chemists. From it we learn that the total quantity of albumen in blood amounts to nearly one-fifth of the whole; that the fibrin forms about $\frac{3}{1000}$ths, while the hæmatosine, or *pure* colouring matter, forms little more than $\frac{2}{1000}$ths of the mass. We have already seen that this yields $\frac{7}{100}$ths of its weight of metallic iron; whence we infer that the proportion of iron in blood is no more than about $\frac{1}{7000}$th. Le Cann makes it $\frac{1}{4405}$, (p. 65;) but, allowing for the decimals, he appears to have calculated for the proportion of oxide of iron, and not of the pure metal. At the same time we fully agree with him that no great importance is to be attached to calculations of this sort; and the less so, since we do not find that there is much agreement in the reports of any two observers. Still, by showing how minute the quantity of metal is, they set at rest two points: they refute the exaggerated statements of the older chemists, and they serve to establish that the colour of the blood cannot depend upon the mere presence of this metal.

In regard to *Sex:* it is found that, while the proportion of albumen is about the same, the blood of the male contains less water and more globules than the blood of the female. We are to understand by the albumen here, and in all future observations, that portion only which enters into the composition of serum, and not that which has been described as forming part of the globules. The chief modifications which the blood undergoes in persons of different temperaments and ages are observed to relate chiefly to the relative proportions of serum and globules; the quantity of albumen being subject to little variation. Thus, the serum is in less and the globules are in greater proportion in individuals of a sanguine than in those of a lymphatic temperament; in adults than in infants or old persons; in those who take much nourishment than in those who take little. Thus the author remarks, "the proportion of globules in the blood would seem to be an index of the vital energy." (p. 69.) So again: "By a singular coincidence, every cause that tends to diminish the mass of blood in the body tends at the same time to diminish the relative proportion of globules, by increasing the quantity of water; so that those causes which diminish the fulness of the vessels at the same time impoverish the blood, and render it more fluid. Uterine hemorrhage in the female, and copious bleeding in either sex, are well known to have this effect." (ib.)

The author next describes the differences between arterial and venous blood, and he arranges in order the observations of different chemists, which are in some instances of the most conflicting character. By sifting the statements of some, and testing the correctness of others by experiment, he comes to the conclusion that the following are the only appreciable differences between the two kinds of blood:

"*Arterial* blood is of a brighter vermilion colour, and possesses a stronger odour, than *venous.* It has a greater tendency to coagulate, furnishes a larger and firmer clot; facts which show that it has a greater proportion of globules and less serum. It contains more fibrin, and the solid matter being in greater proportion, its density is higher than that of venous blood. The albumen, saline and fatty matters, are about equal in the two kinds of blood. Arterial blood is richer in oxygen, in proportion to its carbonic acid, than venous; and, by ultimate analysis, it yields less combined carbon and more combined oxygen." (p. 86.)

The properties of the blood of the *capillary* system are given on the authority of Pallas; of that of the vena portæ, on the authority of Prévost and Dumas; while the characters of placental blood are derived from the observations of Denis. M. Le Canu here presents us with nothing original. The blood of the capillaries was not found to differ sensibly from that of the veins; and, although the blood of the vena portæ seemed to offer a smaller proportion of globules, yet this was only in comparison with the *mean average* of venous blood: the quantity of globules was within the ordinary range of variation of healthy blood. The blood of the placenta Denis found to contain less water and more globules. It is singular that the blood of the fœtus should differ in the same respects from that of the mother; for the proportion of globules in fœtal blood is far greater than in that of the mother. This is precisely the change which the lungs work after birth; and it is a proof, therefore, that the placenta, in the unborn child, really occupies the place of lungs.

We now arrive at the last part of the work, namely, that which refers to the changes which take place in the blood under certain *pathological* states of the system. This is obviously the end to which all researches on the blood ought to be directed; for our knowledge of the chemical properties of this fluid will be valueless, unless made auxiliary to the purposes of medical practice. We wish we could look forward to the realization of the anticipations of the author, as to the good likely to be wrought on medicine by the pursuit of these enquiries; but we fear a very long period must elapse before this end will be attained: nevertheless, we willingly agree with him that, when we compare our knowlege of this important liquid with that which existed but half a century since, we have indeed reason to congratulate ourselves on having made more than one step towards the desired object. The following observations are very just:

"Our knowledge of the pathological changes which take place in the blood must be necessarily imperfect, until we know exactly, and in the minutest details, the composition of normal or healthy blood. At present we are not acquainted with all the immediate principles of the blood in health: for instance, we know nothing of those numerous substances commonly confounded under the name of *extractive matters;* we cannot determine exactly the proportions even of those principles with which we are best acquainted; and, lastly, there is not a single principle in the blood which may not undergo important changes, without these becoming appreciable in analysis." (*Le Canu,* p. 93.)

We should recommend M. Denis to adopt the philosophical caution implied in these sentiments, and not suppose, as he seems to do, that he has left future chemists nothing to occupy themselves with in relation to this subject. For instance, we are told by him "that chemical experiments are everywhere at hand to assist us, and that these must take precedence of all explanations," (p. 180;) while, immediately afterwards, he furnishes us with thirteen conclusions, in as many paragraphs, which, for crude hypothesis and hasty generalization, we have seldom seen surpassed. It is strange that one generation will not profit by the errors of another. We laugh at the false reasoning of the older chemists, but we do not hesitate to plunge into the very system which misled them. It is fortunate, however, that, by a strictly inductive method of research, it is as easy to expose such absurdities as it is to invent them. We shall dismiss this gentleman's "new experiments" on the blood with one quotation, showing the application of his views to therapeutics. It appears that, among other cases, he has endeavoured to cure the croup *on chemical principles*.

"The croup," he says, "consists physically in a false fibrinous membrane lodged in the trachea. M. Denis conceived that it might be attacked directly by the introduction of substances exerting a solvent action over it, and indirectly by introducing into the blood, through the medium of absorption, other substances possessing a similar power. He asserts that he has *successfully* employed for this purpose salts resembling those of the blood." (*Denis*, p. 184.)

But to return to the more cautious views of M. Le Canu. We do not find that the author has advanced much that is original on the pathology of the blood; but he has carefully collected, arranged, and commented on the statements of other experimentalists respecting its abnormal conditions in numerous diseases. He presents us, indeed, here with a good summary of all that is known on this interesting subject.

The menstrual secretion appears to differ from blood in containing more water, less albumen and globules, and an admixture of variable proportions of mucus.

In inflammatory affections, the quantity of globules increases; or, in other words, the proportion of water is diminished.

Dr. Rollo announced, now many years since, that in diabetes the blood contained sugar; but the researches of numerous able experimentalists have rendered it probable that he was deceived. Vauquelin operated on the blood of a diabetic patient, whose urine contained one-seventh of sugar, without finding a trace. The only well-ascertained fact appears to be, that in this disease there is a diminution of the proportion of globules.

The state of the blood in icterus has given rise to considerable discussion. Some have contended that this liquid always contains bile; others that it does not contain any; and a third class of pathologists have admitted, a *mezzo termine*, that it receives only the colouring principle of that secretion. This last opinion appears to be correct: for, while no one has succeeded in detecting all the elements of the bile in the blood of icteric patients, many have found in it yellow, green, and blue colouring principles, resembling, if not identical with, those present in bile. Le Canu observed that the globules were also in much less proportion in these cases. But we must not consider it positively established that the bile never finds its way into the blood. How are we to discover all the complex principles of the bile, more especially picromel, in a still more complex liquid like the blood? There are assuredly no tests at hand to assist us; and, even if there were, it is more than probable, from an experiment of Thénard's, that they would not be available. This distinguished chemist could not find a trace of bile in the blood of an animal, into the veins of which a large quantity had been purposely injected. It had probably been decomposed or altered in the round of circulation. This preliminary experiment should be tried by all those who undertake the pathological analysis of the blood. There is no doubt that the bile has been pronounced, in many instances, to have been present both in the blood and urine, merely from the circumstance of a yellow colouring principle, capable of being affected by mineral acids, like that of bile, having been found in those liquids: but it is quite certain that the discovery of this colouring matter in either case is no proof of the presence of the whole hepatic secretion.

The author had frequent opportunities of examining the blood in malignant cholera, and his results correspond with those obtained by good observers in most countries where this scourge has prevailed. Leaving out trifling differences, which may have depended on purely accidental causes, the most striking and constant deviation appears to have been that the solid matters were in very considerable proportion. At the time that the cholera was most severe in Paris, the following results were obtained from the analysis of the blood of three persons labouring under it.

| | 1st An. | 2d An. | 3d An. |
|---|---|---|---|
| Solid matters (globules) . . | 340 | 251 | 520 |
| Water (serum) . . . . . | 660 | 749 | 480 |
| | 1000. | 1000. | 1000. (p. 106.) |

By this it will be seen that the solid matters were in some instances twice as great as in healthy blood; a condition which did not arise from any increase in the solid, but from a diminution in the liquid contents of the vessels. We agree with the author in thinking that this state of the blood might, perhaps, be made to serve as a criterion of the nature of the malady. The intestinal discharges seem to acquire what the blood loses: these have a powerfully alkaline reaction, and contain albumen with extractive matters, resembling those of the blood.

In typhus fever, the author has found the globules to be below the mean, and even below the minimum proportion in healthy blood. Bonnet observed that hydrosulphuret of ammonia was present in these cases; but this may have depended on the blood having rapidly putrefied after abstraction. This substance is a product of putrefaction; and it is hardly likely that it should be formed within the blood while circulating through the living vessels.

In disease of the heart, the quantity of serum was found to be large, and the clot small. Repeated venesection seemed to have the effect of increasing the proportion of globules.

After the discovery of iron in the blood, it was supposed that the pallor peculiar to chlorosis was due to a diminution in the quantity of that metal: and this seems to have been the foundation of the use of ferruginous preparations in its treatment. Le Canu found that chlorotic blood contained much less than its healthy proportion of globules, and consequently less iron: but the disease cannot be owing to this deficiency, since the same diminution is met with in other and widely different diseases. Other experimentalists have corroborated these results.

In some pathological conditions, as in diabetes, dropsy, peritonitis, nephritis, and hepatitis, the blood has been observed to assume a creamy appearance, just as if milk had been mixed with it. This appearance has been proved by the author and other chemists to be entirely due to the suspension of an excess of animal fat or oil in the serum, and not to casein, as it was formerly supposed. There is at the same time a considerable deficiency, or almost entire absence, of red colouring matter.

Such is an outline of what we believe to be the principal facts connected with the healthy and morbid condition of the blood, so far as the observations of chemists have yet extended. The chief fluctuations in disease relate to the increase or diminution of the globules; and we cannot hesitate to assent to the proposition of M. Le Canu, that many morbid states in the system may be due to insidious changes in the nature and proportion of the constituents of this important liquid. Without adopting the extreme views of humoral pathologists, there is nothing to prevent us supposing that fatal impressions on the system may also operate by altering the blood. Many poisons may in this manner destroy life, although chemistry has not yet succeeded in establishing their presence in this liquid.

We shall conclude our notice of M. Le Canu's thesis by saying that it reflects the highest credit on the author. There is much that is original; and this, together with what he has compiled from numerous sources, forms, in our opinion, the best modern history of the blood.

---

